# Partograph utilization as a decision-making tool and associated factors among obstetric care providers in Ethiopia: a systematic review and meta-analysis

**DOI:** 10.1186/s13643-020-01505-4

**Published:** 2020-11-03

**Authors:** Asteray Assmie Ayenew, Biruk Ferede Zewdu

**Affiliations:** 1 Department of Midwifery, College of Medicine and Health Sciences, Bahir Dar University, Bahir Dar, Ethiopia; 2 Department of Orthopedics, College of Medicine and Health Sciences, Bahir Dar University, Bahir Dar, Ethiopia

**Keywords:** Partograph utilization, Obstetric care providers, Systematic review and meta-analysis, Ethiopia

## Abstract

**Background:**

Globally, a total of 13.6 million women have died due to maternal causes from 1990 to 2015. Majority of these deaths occurred in resource-limited countries. Among the causes of these deaths, obstructed and prolonged labor covers the highest percentage, which could be prevented by cost-effective and affordable health interventions like partograph use. Therefore, this systematic review and meta-analysis aimed to assess the level of partograph utilization among obstetric care providers and its associated factors in Ethiopia.

**Method:**

For this review, we used the standard PRISMA checklist guideline. Different online databases were used for the review: PubMed/Medline, Google Scholar, EMBASE, Cochrane Library, HINARI, WHO Afro Library Databases, and African Online Journals. Based on the adapted PICO principles, different search terms were applied to achieve and access all the essential articles. Microsoft Excel was used for data entry and Stata version 11.0 (Stata Corporation, College Station, TX, USA) for data analysis.

**Result:**

Nineteen studies were included in this systematic review and meta-analysis with a total of 6237 obstetric care providers. The overall pooled prevalence of partograph utilization was 59.95% (95% CI 46.8–73.09, *I*^2^ = 99.4%, *P* < 0.001). Being in midwifery profession (adjusted odds ratio (AOR) 3.97; 95% confidence interval (CI) 2.63–5.99, *I*^2^ = 28.8%, *P* = 0.198), presence of supervision (AOR = 3.21; 95% CI 2.22–4.66, *I*^2^ = 0.0%, *P* = 0.742), Basic Emergency Obstetric and Newborn Care (BEmONC) training (AOR = 2.90; 95% CI 2.19–3.84, *I*^2^ = 36.9%, *P* = 0.13), knowledge of partograph (AOR = 2.5; 95% CI 1.6–3.8, *I*^2^ = 64.58%, *P* = 0.024), on-the-job refresher training on partograph (AOR = 5.7; 95% CI 2.5–12.9, *I*^2^ = 87.8%, *P* < 0.001), favorable attitude (AOR = 2.12; 95% CI 1.48–3.04, *I*^2^ = 0.0%, *P* = 0.58), and working at health center (AOR = 3.50; 95% CI 2.49–4.92, *I*^2^ = 49.1%, *P* = 0.08) were the determinant factors for partograph use among obstetric care providers in Ethiopia.

**Conclusion:**

The overall pooled prevalence of partograph utilization among obstetric care providers was low. Therefore, supportive supervision, providing Basic Emergency Obstetric and Newborn Care training, on-the-job refresher training on partograph, and promoting midwifery profession are strongly recommended to increase the use of partograph.

**Supplementary Information:**

**Supplementary information** accompanies this paper at 10.1186/s13643-020-01505-4.

## Background

Globally, a total of 13.6 million women have died due to maternal causes from 1990 to 2015. Of all the deaths, 99% were in developing countries with 546 per 100,000 live births, and sub-Saharan Africa only accounts 66% of deaths [1]. Additionally, there is a staggering evidence that peripartum fetal mortality and morbidity are directly related to the labor abnormalities like asphyxia, birth injuries, low Apgar scores (Appearance, Pulse, Grimace, Activity, and Respiration), and intrapartum or postpartum deaths. About 97% of all reported neonatal deaths occur in less developed countries. Of these, majority are a direct consequence of labor complications [2]. In Ethiopia, the tragedy of maternal and neonatal mortality is stagnant so far, in spite of the apparent commitment by stakeholders. In 2016, maternal mortality accounted for 412 per 100,000 live births and neonatal mortality was 29 per 1000 live births [3].

According to the World Health Organization (WHO), one of the key important requirements for averting these deaths is the provision of care by a skilled birth attendant before, during, and after childbirth [4]. Skilled birth attendant care needs to be available across all levels of the health system in order to reduce the delays for a referral to a higher care level if problems are expected to arise or do arise during labor. Thus, partograph is also used in conjunction with this intervention [5]. The partograph is a graphical record of the progress of labor and relevant details of the mother and the fetus. It has action and alert lines to stimulate commencement of additional interventions by a skilled birth attendant monitoring the progress of labor [6].

Partograph is an effective tool to monitor the progress of labor. When used effectively, it prevents obstructed labor, which is a leading cause of maternal and neonatal mortality, especially in developing countries [7–9]. Globally, it is estimated that obstructed labor occurs in 5% of pregnancies and accounts for an estimated 8% of maternal deaths [10–12], whereas the prevalence of obstructed labor is 47% in Ethiopia and accounts for 9% of the total maternal death [3].

Friedman in 1954 studied the natural course of human labor and proposed a new way of plotting progress of labor in the first stage, against time, progress faster at the rate of 1.3 cm/h in primigravida and 1.5 cm/h in multigravida. Friedman devised a graph of labor depicting cervical dilatation and descent of fetal head in a graphical manner against time; this was known as “the Friedman’s curve.” The graph is a sigmoid curve divided into latent and active phase of labor [13]. The curve later became the basis of the modern partograph that is in clinical use today. The World Health Organization launched the partogram in 1987 as a safe motherhood initiative following a multi-center trial [14]. The first WHO partograph or “composite partograph” covers a latent phase of labor of up to 8 h and an active phase beginning when the cervical dilatation reaches 3 cm. The active phase is depicted with an alert line and an action line, drawn 4 h apart on the partograph. This partograph is based on the principle that during active labor, the rate of cervical dilation should not be slower than 1 cm/h. Since a prolonged latent phase is relatively infrequent and not usually associated with poor perinatal outcome, the usefulness of recording the latent phase of labor in the partograph has been questioned. Moreover, differentiating the latent phase from false labor is often difficult [8]. To alleviate these disadvantages, a modified WHO partograph was introduced and incorporated removal of the latent phase and defined the beginning of the active phase at 4 cm cervical dilatation instead of 3 cm [15].

The World Health Organization recommends the universal utilization of the partograph during labor for routine monitoring of labor, and helps the health care provider in identifying slow progress in labor, and to make better decisions for the diagnosis and management of prolonged and obstructed labor [16, 17].

Moreover, partograph serves as an “early warning system” and assists in early decisions on transfer, intervention decisions in hospitals, and ongoing evaluation of the effect of interventions to prevent maternal deaths caused by prolonged labor. It has been promoted by the World Health Organization as the “gold” standard for assessing the progress of labor especially for low resource countries like Ethiopia [18, 19].

For pregnant women, obstructed labor remains an important cause of not only maternal death but also short- and long-term disability like obstetric fistula, uterine rupture, uterine prolaps, nerve damage, incontinence, puerperal sepsis, postpartum hemorrhage, and infertility from hysterectomy [20]. This can be prevented by accessing skilled delivery services such as plotting partograph during the progress of labor [14, 21]. Therefore, this systematic review and meta-analysis aimed to estimate the pooled prevalence of partograph use among obstetric care providers and its determinant factors in Ethiopia.

## Methods

This systematic review and meta-analysis was conducted to estimate the national use of partograph and its associated factors among obstetric care providers in Ethiopia. We used the Preferred Reporting Items for Systematic Reviews and Meta-Analyses (PRISMA) checklist guideline [22] (Additional file 1).

### Searching strategy

First, the PROSPERO database and Database of Abstracts of Reviews of Effects (DARE) (http://www.library.UCSF.edu) were searched to check whether published or ongoing projects exist related to the topic. The literature search strategy, selection of studies, data extraction, and result reporting were done in accordance with the Preferred Reporting Items for Systematic Reviews and Meta-Analyses (PRISMA) guidelines [23]. We searched PubMed, Google Scholar, EMBASE, Cochrane Library, HINARI, WHO Afro Library Databases, and African Online Journal databases for all available studies using the following terms: “Partograph utilization,” “knowledge on partograph,” “labor,” “on-the-job refresher training on partograph,” “obstetric care providers,” “midwives,” “decision making tool,” “attitude towards partograph,” “labor monitoring,” “first stage of labor,” “health care providers,” “health institutions,” “childbirth,” “factors,” “determinants,” “health institutions,” “intrapartum monitoring,” “components,” “partograph,” and “Ethiopia.” The search string was developed using “AND” and “OR” Boolean operators. Searching terms were based on adapted PICO principles to search through the above-listed databases to access all the relevant articles. For unpublished studies, the official websites of Ethiopian’s university research repository online library (University of Gondar and Addis Ababa University) were used.

### Inclusion and exclusion criteria

In this systematic review and meta-analysis, we included all observational studies (cross-sectional, case-control, and cohort studies) conducted in Ethiopia and only in English language. Additionally, studies that reported prevalence and/or risk factors, the outcome was partograph utilization as a decision-making tool, and participants of obstetric care providers and both published and unpublished studies at any time were included. However, studies available only as abstract with unclear outcomes, commentaries, editorials, reviews, and qualitative studies were excluded.

### Quality assessment

After collecting the findings from all databases, the articles were exported to Microsoft Excel spreadsheet. Two authors (AAA and BFZ) independently extracted the data and reviewed the screened and eligible articles. Any disagreement was handled by the two reviewers (AAA and BFZ). Finally, a consensus was reached between the two authors through discussion. The methodological quality of each study (sampling strategy, response rate, and representativeness of the study), comparability, and outcome were checked using the NOS tool. Newcastle-Ottawa Quality Assessment Scale (NOS) for cross-sectional and case-control studies was used to assess the methodological quality of a study and to determine the extent to which a study has addressed the possibility of bias in its design, conduct, and analysis [24]. All the included articles scored (NOS) 7, and more can be considered as “good” studies with low risk (Additional file 2).

### Outcome of measurement

This review has two main outcomes. Partograph utilization among obstetric care providers to monitor the progress of labor and feto-maternal condition was the primary outcome of the study, whereas associated factors for partograph utilization among obstetric care providers were the second outcome variables. The odds ratio was calculated for the common risk factors of the reported studies. The most common associated factors included in this systematic review and meta-analysis were midwifery profession, presence of supervision, Basic Emergency Obstetric and Newborn Care (BEmONC) training, knowledge of partograph, on-the-job refresher training on partograph, favorable attitude towards partograph, and working at the health center.

### Data extraction

Microsoft Excel (2016) and Stata version 11.0 (Stata Corporation, College Station, TX, USA) software were used for data entry and analysis, respectively. Two authors (AAA and BFZ) independently extracted all the important data using a standardized JBI data extraction format. The inter-rater agreement between authors for study inclusion, data extraction, and methodological quality will be assessed using Cohen’s kappa coefficient (values ≤ 0 as indicating no agreement and 0.01–0.20 as none to slight, 0.21–0.40 as fair, 0.41– 0.60 as moderate, 0.61–0.80 as substantial, and 0.81–1.00 as almost perfect agreement) [25]. Substantial agreement between reviewers, i.e., Cohen’s kappa coefficient > 0.60, was accepted. Any disagreement between reviewers was resolved through discussion, and then, consensus was reached. During data extraction, name of the author, sample size, publication year, study design, prevalence, response rate, population outcome, study site, and different contributing factors were included. Moreover, prevalence of partograph use with 95% CI and associated factors were collected [26].

### Statistical analysis

As the test statistic showed significant heterogeneity among studies (*I*^2^ = 99.4%, *P* < 0.05), the random-effects model was used to estimate the DerSimonian and Laird pooled effect [27]. Cochran’s *Q* chi-square statistics and *I*^2^ statistical test were conducted to assess the random variations between primary studies [28]. In this study, heterogeneity was interpreted as an *I*^2^ value of 0% = no heterogeneity, 25% = low, 50% = moderate, and 75% = high [29]. In case of high heterogeneity, subgroup analysis and sensitivity analyses were run to identify possible moderators of this heterogeneity. Potential publication bias was assessed by visually inspecting funnel plots and objectively using Egger’s test (i.e., *P* < 0.05) [30]. To account for any publication bias, we used the trim-and-fill method, based on the assumption that the effect sizes of all the studies are normally distributed around the center of a funnel plot. The meta-analysis was performed using the Stata version 11.0 (Stata Corporation, College Station, TX, USA) software. Finally, for all analyses, *P* < 0.05 was considered statistically significant.

## Results

### Study selection and data extraction

**Fig. 1:**
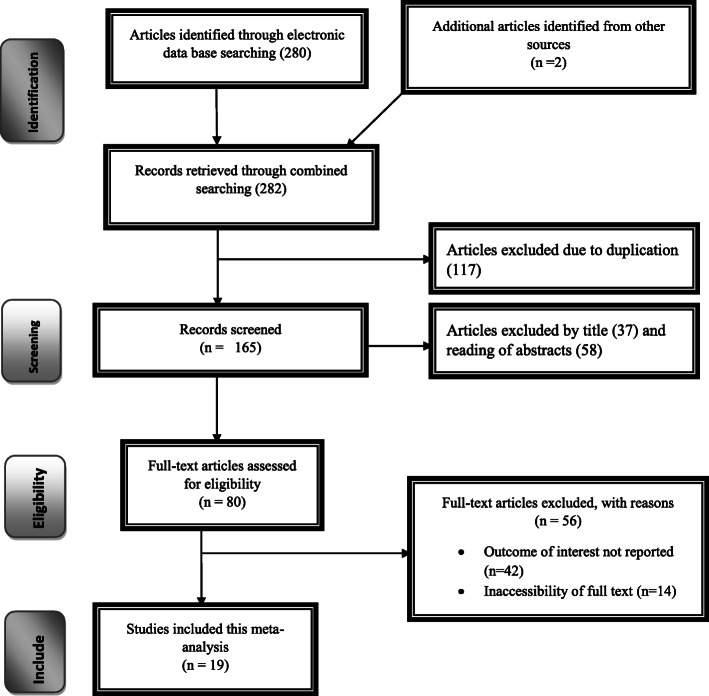
Flow chart of study selection for systematic review and meta-analysis of partograph utilization as a decision-making tool among obstetric care providers in Ethiopia

The search strategy identified 80 articles from PubMed, 60 articles from Google Scholar, 45 articles from Cochrane Library, 10 articles from African Journals Online, 7 articles from Ethiopian’s university online library, and 5 articles by manual search. Of them, 134 were excluded due to duplication, and 35 through review of titles and abstracts. Additionally, 31 full-text articles were excluded for not reporting the outcome variable and other reasons. Finally, 19 were included to the prevalence and/or associated factor analysis on partograph use (Fig. 1).

### Study characteristics

Different factors such as midwifery profession, presence of supervision, Basic Emergency Obstetric and Newborn Care (BEmONC) training, knowledge of partograph, on-the-job refresher training on partograph, favorable attitude towards partograph, and working at the health center were included in this study. Nineteen cross-sectional studies with a total of 6237 obstetric care providers were included in this review. All of the included articles were facility-based study setting. Regarding the study area, six of the studies were conducted at SNNPR (South Nation Nationalities and People Representative), four in Tigray, and three each in Amhara and Oromia, respectively (Table 1).

**Table 1:** Descriptive summary of nineteen included studies in the systematic review and meta-analysis

Author (year of study) (reference number)	Sample size	Response rate (%)	Study region	Prevalence (95% CI)	NOS quality of score
Fantu et al. (2012) [31]	381	88	Amhara	29 (24–33)	9
Habtamu et al. (2017) [32]	224	90.2	Oromia	89 (85–93)	9
Wakeshe et al. (2015) [33]	266	97.4	Oromia	84 (80–88)	9
Negash et al. (2013) [34]	403	94.5	Amhara	40 (35–45)	8
Haymanot et al. (2015) [35]	441	98	Addis Ababa	92.6 (90–95)	9
Tesfay et al. (2017) [36]	220	90	Tigray	73 (67–79)	9
Desalegne et al. (2015) [37]	273	100	Amhara	53 (48–60)	9
Kidist et al. (2016) [38]	300	93.3	SNNP	51 (45–57)	8
Kidest et al. (2016) [39]	442	99	SNNP	73 (68–78)	9
Markos et al. (2014) [40]	401	91	SNNP	70 (66–75)	9
Engida et al. (2012) [41]	202	96.5	Addis Ababa	57 (50–64)	8
Sena et al. (2012) [42]	340	80.6	Oromia	6.9 (4–10)	9
Gutema et al. (2015)	309	89	SNNP	54 (48–59)	9
Daniel et al. (2016) [43]	127	100	SNNP	26 (18–34)	9
Haftom et al. (2015) [44]	233	93	Tigray	57 (51–64)	9
Guesh et al. (2018) [45]	414	98.1	Tigray	83 (31–43)	9
Haile et al. (2019) [46]	436	95	SNNP	55.4 (2–9)	9
Tesfay et al. (2019) [36]	220	98	Tigray	73.3 (21–32)	9
Azeb et al. (2017) [47]	605	98.1	Addis Ababa	69 (36–45)	9

### Partograph use among obstetric care providers in Ethiopia

**Fig. 2:**
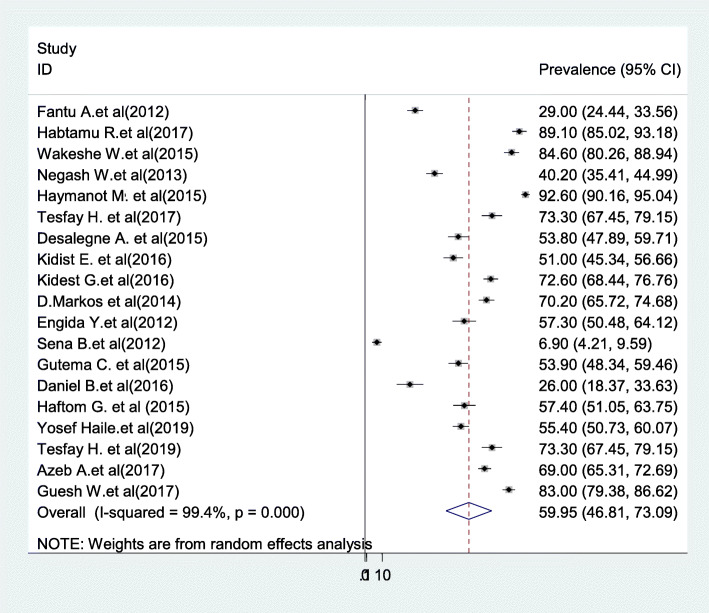
Forest plot for the prevalence of partograph use among obstetric care providers in Ethiopia, 2020

The overall pooled prevalence of partograph is presented with a forest plot (Fig. 2). Therefore, the national estimated prevalence of partograph use among obstetric care providers in Ethiopia was 59.95% (95% CI 46.8–73.09, *I*^2^ = 99.4%, *P* < 0.001).

### Publication bias

**Fig. 3:**
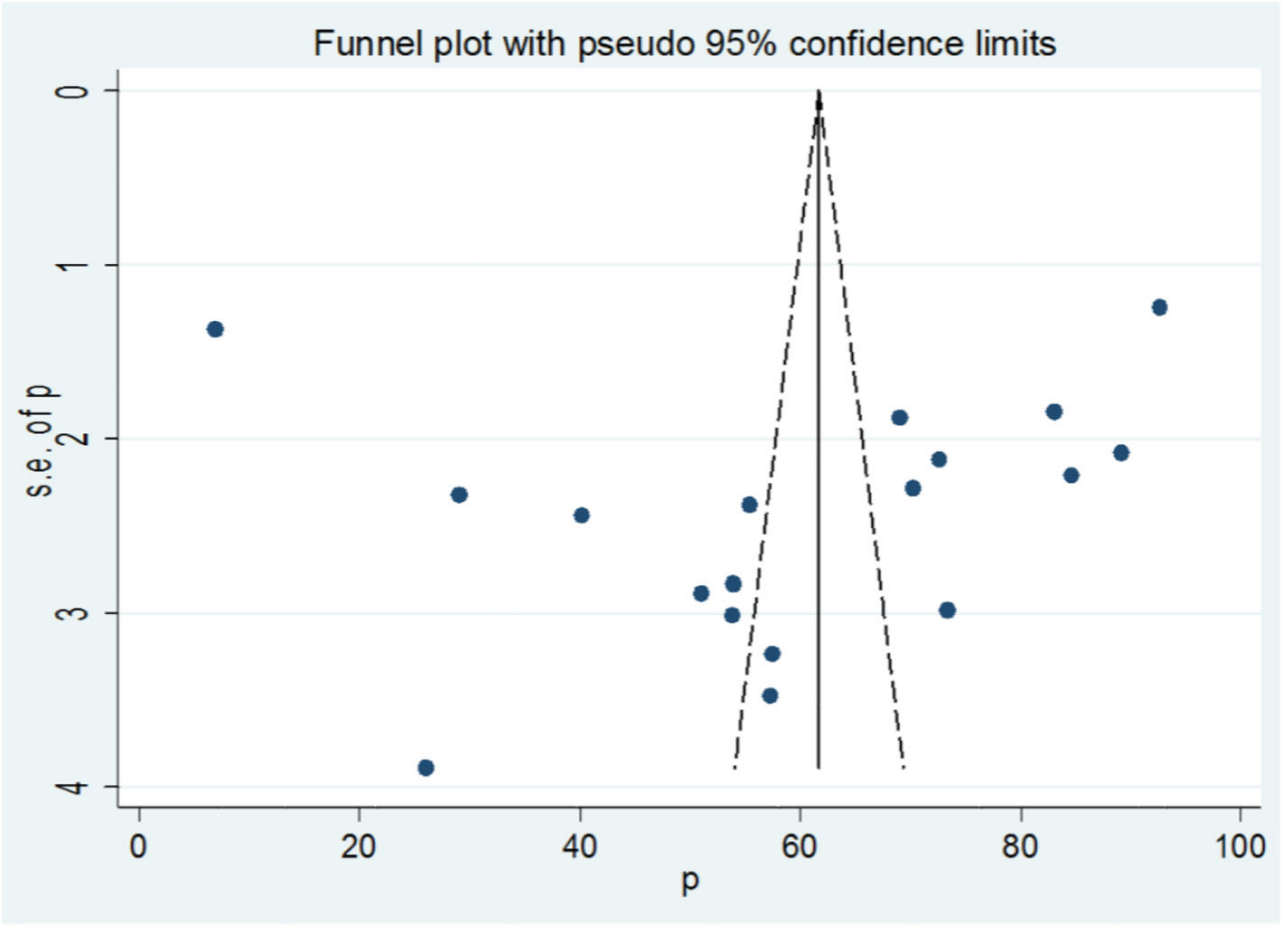
Funnel plot with 95% confidence limits of the pooled prevalence of partograph use among obstetric care providers in Ethiopia, 2020

The funnel plot was assessed for asymmetry distribution of prevalence of partograph use among obstetric care providers by visual inspection (Fig. 3). Egger’s regression test showed a *P* value of 0.759 with no evidence of publication bias.

### Sensitivity analysis

**Fig. 4:**
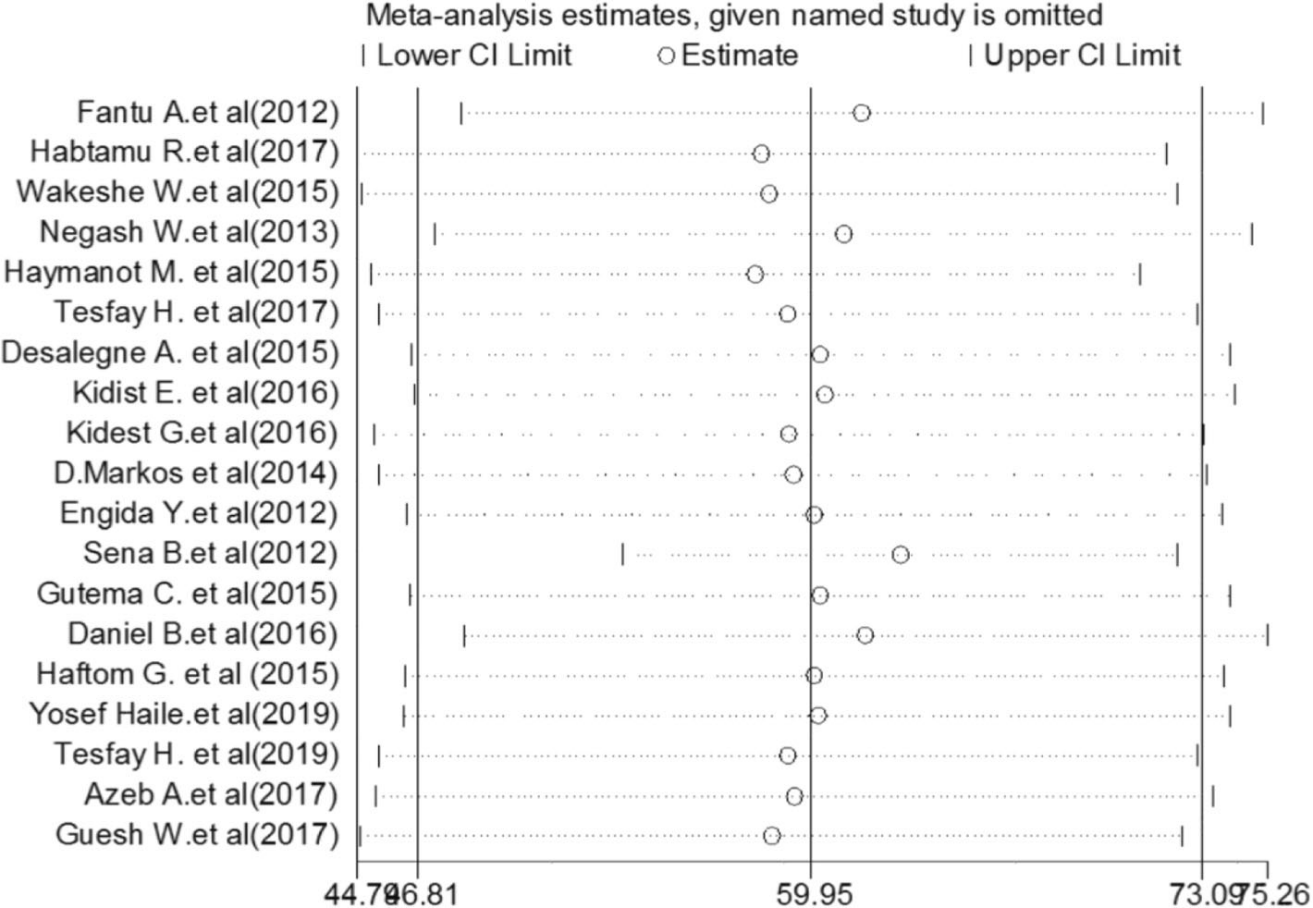
Sensitivity analysis of the pooled prevalence of partograph use among obstetric care providers in Ethiopia

This systematic review and meta-analysis showed that the point estimate of its omitted analysis lies within the confidence interval of the combined analysis. Therefore, trim-and-fill analysis was not further computed (Fig. 4).

### Subgroup analysis

**Fig. 5:**
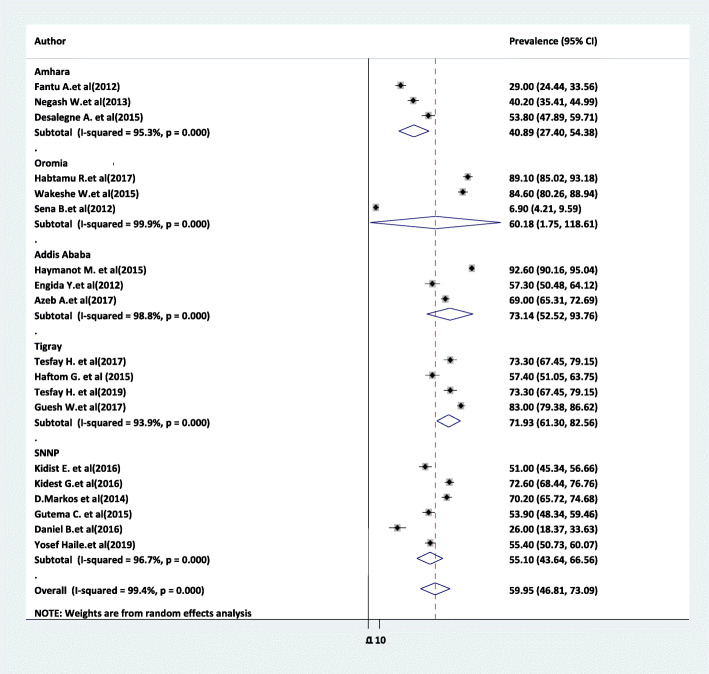
Subgroup analysis of the pooled prevalence of partograph use among obstetric care providers based on the study region

**Fig. 6:**
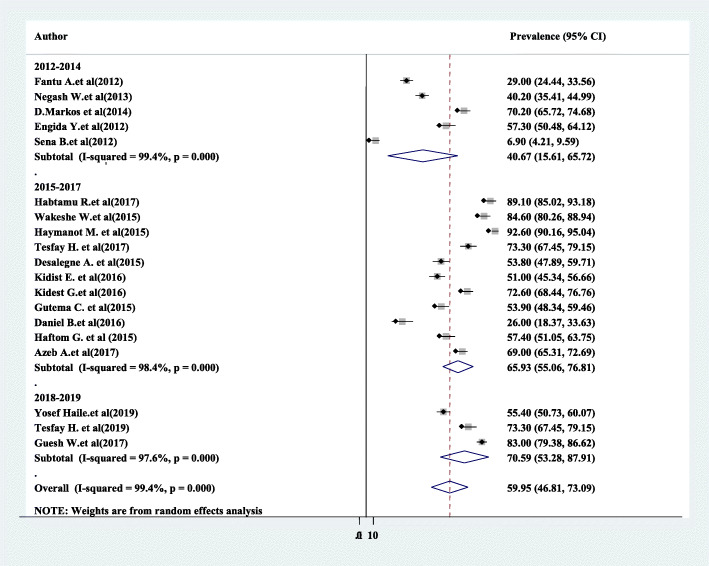
Subgroup analysis of the pooled prevalence of partograph use among obstetric care providers based on the year of study

Subgroup analysis was employed with the evidence of heterogeneity. In this study, the Cochrane *I*^2^ statistic was 99.4%, *P* < 0.001, which showed the evidence of marked heterogeneity. Therefore, subgroup analysis was done using the study region and year of study. As a result, the use of partograph was highest in Addis Ababa 73.4%, whereas 70.95% in the study conducted between 2018 and 2019 (Figs. 5 and 6).

### Determinants of partograph utilization in Ethiopia

The association between midwifery profession, presence of supervision, Basic Emergency Obstetric and Newborn Care (BEmONC) training, attitude, knowledge of partograph, on-the-job refresher training on partograph, favorable attitude towards partograph, and working at health centers with partograph use was carried out.

In this meta-analysis, to identify the associated factors, eight articles were used for midwifery profession [31–35, 40, 43, 48], five for knowledge of partograph [33, 34, 37, 46, 47], three for attitude [34, 35, 46], five for on-the-job refresher training on partograph [31, 35, 40, 46, 47], seven for BEmONC training [31, 33–37, 44], four for presence of supervision [31, 32, 40, 43], and six for working at health center [35, 37, 38, 41, 46, 48].

Obstetric care providers who were midwives were 3.97 times more likely to use partograph as a decision-making tool. Those obstetric care providers who received BEmONC training were 2.9 times more likely to use partograph. The odds ratio of on-the-job refresher training on partograph to use partograph was 5.7. Obstetric care providers who supervised were 3.21 times more likely to use partograph. Additionally, obstetric care providers who had a good knowledge of partograph were 2.5 times more likely to use partograph.

Obstetric care providers who had a favorable attitude towards partograph utilization were 2.12 more likely to utilize partograph as a decision-making tool. Moreover, those obstetric care providers working at the health center were 3.5 times more likely to use partograph (Table 2).

**Table 2:** Descriptive summary of determinant factors for partograph utilization among obstetric care providers

Variable name	No. of included studies	OR (95% CI)	Overall (*I*-squared, *P* value)
Midwifery profession	8	3.97 (2.63–5.99)	28.8%, *P* = 0.198
Presence of supervision	4	3.21 (2.22–4.66)	0.0%, *P* = 0.742
Emergency Obstetric and Newborn Care training	7	2.90 (2.19–3.84)	36.9%, *P* = 0.134
Knowledge of partograph	5	2.46 (1.60–3.77)	64.5%, *P* = 0.024
Attitude towards partograph	3	2.12 (1.48–3.04)	0.0%, *P* = 0.573
On-the-job refresher training on partograph	5	5.66 (2.48–12.92)	87.8%, *P* = 0.000
Working at health center	6	3.50 (2.49–4.92)	49.1%, *P* = 0.08

## Discussion

The use of partograph in this review ranged from 6.9 to 92%. The highest use of partograph use was from Addis Ababa [35] while the lowest one was from Oromia region [42]. The purpose of this review was to assess the pooled prevalence and associated factors of partograph use by reviewing the finding of available studies. The pooled prevalence of partograph use in Ethiopia was 59.9%.

The use of partograph for all laboring mothers is recommended by the WHO as a means to monitor and record maternal and fetal well-being, as it can identify maternal or fetal distress and abnormalities in the progress of labor that require further action, including referral. This can reduce complications from prolonged labor for the mother (obstetric fistula, postpartum hemorrhage, sepsis, uterine rupture, and its sequelae) and for the infant (death, anoxia, and infections) [49–51]. Thus, the use of partograph in this study was low.

The findings of the current study is lower than studies in South Africa [52] which accounted for 79.4%, Ghana [53] 87%, Gambia [54] 78%, and Uganda [55] 69.9%. The differences between these findings might be due to difference in level of knowledge of obstetric care providers and different strategies in partograph implementation; as in Ghana, obstetric care providers received specific training in the use of partograph [56], 83.8% trained in South Africa [52], and the application of improving partograph use in Uganda through training, coaching, and mentoring [57]. Moreover, in the studies of Gambia, Uganda, and South Africa, the participants were only midwives and doctors by profession, whereas this study included all obstetric care providers including nurses, health extension workers, public health officers, and IESOs.

The prevalence of partograph use in this study is higher than the study done in Rwanda [58] 41.22%, Cameroon [59] 32.4%, and Nigeria [50] 37.5%. The possible reason might be the Federal Ministry of Health has set targets and working for facility delivery coverage at 90% and to enable all health centers to use partograph, and to provide all BEmONC functions [60].

The finding of this meta-analysis revealed that midwives were 3.97 times more likely to use partograph compared to other obstetric care providers. This result agrees with studies conducted in Nigeria [50] and South Africa [61]. This might be justified by midwives to have more chances of being assigned in labor wards and consequently received training on partograph, which might in turn have improved their knowledge, skill, and attitude to use partograph than others.

In this study, obstetric care providers who supervised were 3.21 times more likely to utilize partograph than their counterparts. The possible reason could be due to the availability of well-designed and coordinated programs like the strength of mentorship, and supportive supervision of obstetric care providers may affect the use of partograph. Thus, effective utilization of obstetric care provider’s knowledge advancement through refresher training, including practical demonstration, supportive supervision, and on-site partograph audits by trained supervisors, should also be prioritized [55, 62, 63].

Obstetric care providers who received Basic Emergency Obstetric and Newborn care training were 2.9 times more likely to use partograph than who did not receive. This finding is in line with a study done in Malawi [64] and Nigeria [65]. The reason might be receiving Emergency Obstetric and Newborn care training to capacitate obstetric care providers to interpret the components of partograph, to follow best practices during childbirth, and to use partograph as a decision-making tool.

Obstetric care providers who had adequate knowledge on partograph were 2.5 times more likely to utilize partograph than their counterparts. This result was supported by other studies [65, 66]. The possible reason might be the knowledge that enables them to understand what critical progress of labor will occur and decide on alternatives such as referral and caesarian section which encourage obstetric care providers to use partograph as a decision-making tool.

Additionally, obstetric care providers who received on-the-job refresher training on partograph were 5.7 times more likely to use partograph compared to those who did not receive. This result in supported by a study in Tanzania [67]. The possible reason might be health care providers who received on-the-job refresher training on partograph had better knowledge, skills, and confidence about partograph use, which in turn improves its use.

Partograph utilization was 2.12 times higher among obstetric care providers who had a favorable attitude as compared to those who had an unfavorable attitude. This is in agreement with the studies done in Nigeria [50]. The possible reason might be having a favorable attitude towards partograph might come after having knowledge about partograph that may influence partograph use.

According to this study, obstetric care providers who have been working at health centers were 3.5 times more likely to use partograph compared to those who have been working at hospitals. The possible explanation might be the majority of the obstetric care providers working in health centers received on-the-job refresher training on partograph, BEONC training, and frequent supervision as compared to those working in hospitals [68]. The other reason might be health center obstetric care providers use partograph as a guide to take early action, to have adequate evidence, and to refer to higher health institution, which might increase its use.

## Limitation

Since it is the first systematic review and meta-analysis, it is taken as strength. The included articles were restricted to the English language only; this is a limitation of the study as it missed studies published in local languages. This review has not been registered online.

## Conclusion

The overall pooled prevalence of partograph utilization among obstetric care providers in Ethiopia was low. Therefore, supportive supervision, providing Basic Emergency Obstetric and Newborn Care training, on-the-job refresher training on partograph, and promoting midwifery profession are strongly recommended to increase the use of partograph.

## Supplementary Information

**Additional file 1.** PRISMA checklist.

**Additional file 2.** NOS quality score.

## Data Availability

The data sets generated during the current study are available from the corresponding author on reasonable request.

## Abbreviations

AA: Addis Ababa
CI: Confidence interval
EmOC: Emergency obstetric care
NC: Newborn care
OR: Odds ratio
PRISMA: Preferred Reporting Items for Systematic Reviews and Meta-Analyses
SNNP: Southern Nation Nationality and People
WHO: World Health Organization
