# Supplementary material for: Partograph utilization as a decision-making tool and associated factors among obstetric care providers in Ethiopia: a systematic review and meta-analysis
Source: Syst Rev. 2020 Nov 3;9:251. doi: 10.1186/s13643-020-01505-4 (PMC7640697; doi:10.1186/s13643-020-01505-4)
Supplement: Supplementary file 2 — Additional file 2. NOS quality score. [file 13643_2020_1505_MOESM2_ESM.docx]

| **NOS scale for cross sectional studies** | Selection | | | | Comparability | Outcome | | Total score |
| --- | --- | --- | --- | --- | --- | --- | --- | --- |
|  | Representativeness  (1) | Sample size  (1) | Non-respondents  (1) | Ascertainment of the exposure (risk factor)  (2) | The subjects in different outcome groups are comparable, based on the study design or analysis. Confounding factors are controlled (2) | Assessment of the outcome  (2) | Statistical test  (1) |  |
| Fantu A.et al(2012)[29] | * | * | * | ** | * | ** | * | 9 |
| Habtamu R.et al(2017)[30] | * | * | * | * | ** | ** | * | 9 |
| Wakeshe W.et al(2015)[31] | * | * | * | ** | * | ** | * | 9 |
| Negash W.et al(2013)[32] | * | * | * | * | * | ** | * | 8 |
| Haymanot M. et al(2015)[33] | * | * | * | ** | * | ** | * | 9 |
| Tesfay H. et al(2017)[34] | * | * | * | ** | * | ** | * | 9 |
| Desalegne A. et al(2015)[35] | * | * | * | ** | * | ** | * | 9 |
| Kidist E. et al(2016)[36] | * | * | * | * | * | ** | * | 8 |
| Kidest G.et al(2016)[37] | * | * | * | ** | * | ** | * | 9 |
| D.Markos et al(2014)[38] | * | * | * | * | * | ** | * | 9 |
| Engida Y.et al(2012)[39] | * | * | * | ** | * | ** | * | 8 |
| Sena B.et al(2012)[40] | * | * | * | ** | * | ** | * | 9 |
| Gutema C. et al(2015) | * | * | * | ** | * | ** | * | 9 |
| Daniel B.et al(2016)[41] | * | * | * | ** | * | ** | * | 9 |
| Haftom G et al(2015)[42] | * | * | * | ** | * | ** | * | 9 |
| Guesh W.et al(2018)[43] | * | * | * | ** | * | ** | * | 9 |
| Yosef Haile.et al(2019)[44] | * | * | * | ** | * | ** | * | 9 |
| Tesfay H. et al(2019)[34] | * | * | * | ** | * | ** | * | 9 |
| Azeb A.et al(2017)[45] | * | * | * | ** | * | ** | * | 9 |
